# Blood Magnesium, and the Interaction with Calcium, on the Risk of High-Grade Prostate Cancer

**DOI:** 10.1371/journal.pone.0018237

**Published:** 2011-04-25

**Authors:** Qi Dai, Saundra S. Motley, Joseph A. Smith, Raoul Concepcion, Daniel Barocas, Susan Byerly, Jay H. Fowke

**Affiliations:** 1 Vanderbilt Epidemiology Center, Vanderbilt-Ingram Cancer Center, Vanderbilt University Medical Center, Vanderbilt University School of Medicine, Nashville, Tennessee, United States of America; 2 Department of Urologic Surgery, Vanderbilt University Medical Center, Nashville, Tennessee, United States of America; 3 Urology Associates, Nashville, Tennessee, United States of America; Memorial Sloan-Kettering Cancer Center, United States of America

## Abstract

**Background:**

Ionized calcium (Ca) and magnesium (Mg) compete as essential messengers to regulate cell proliferation and inflammation. We hypothesized that inadequate Mg levels, perhaps relative to Ca levels (e.g. a high Ca/Mg ratio) are associated with greater prostate cancer risk.

**Study Design:**

In this biomarker sub-study of the Nashville Men's Health Study (NMHS), we included 494 NMHS participants, consisting of 98 high-grade (Gleason≥7) and 100 low-grade cancer cases, 133 prostate intraepithelial neoplasia (PIN) cases, and 163 controls without cancer or PIN at biopsy. Linear and logistic regression were used to determine associations between blood Ca, Mg, and the Ca/Mg ratio across controls and case groups while adjusting for potential confounding factors.

**Results:**

Serum Mg levels were significantly lower, while the Ca/Mg ratio was significantly higher, among high-grade cases vs. controls (p = 0.04, p = 0.01, respectively). Elevated Mg was significantly associated with a lower risk of high-grade prostate cancer (OR = 0.26 (0.09, 0.85)). An elevated Ca/Mg ratio was also associated with an increased risk of high-grade prostate cancer (OR = 2.81 (1.24, 6.36) adjusted for serum Ca and Mg). In contrast, blood Ca levels were not significantly associated with prostate cancer or PIN.Mg, Ca, or Ca/Mg levels were not associated with low-grade cancer, PIN, PSA levels, prostate volume, or BPH treatment.

**Conclusion:**

Low blood Mg levels and a high Ca/Mg ratio were significantly associated with high-grade prostate cancer. These findings suggest Mg affects prostate cancer risk perhaps through interacting with Ca.

## Introduction

Prostate cancer is the most common non-cutaneous malignancy in Western societies and the second leading cause of cancer death in men [1]. A number of prospective studies have investigated the relationship between calcium and overall prostate cancer risk, with decidedly mixed results [2]–[6]. Several studies also investigating the relationship between calcium intake and the risk of aggressive or clinically relevant prostate cancers have generated both null [4], [6] and positive results [7]–[13]. In contrast, two recent studies found higher serum calcium levels associated with aggressive lesions or fatal prostate cancer [14], [15]. Blood calcium levels are tightly regulated, and only moderately affected by dietary intake of calcium and absorption rate [16]. Thus, one possible explanation for the inconsistencies across study populations is that dietary intake measures of calcium may not accurately reflect the blood calcium concentration to which prostate tissue is exposed.

Magnesium is the second most abundant intracellular cation in the body, involved with over 300 biological activities [17], and calcium and magnesium levels in the body are jointly regulated through a negative feedback system [16], and through competition for intestinal absorption and renal reabsorption [18]. Calcium and magnesium also compete for membrane binding sites within the cell, and previous *in vitro* and *in vivo* studies indicate that magnesium inhibits calcium activity or that magnesium deficiency enhances the physiologic effects of calcium [17], [19], [20].

The 1999–2000 National Health and Nutrition Examination Survey found 79% of U.S. adults have a magnesium intake below the Recommended Dietary Allowance [21]. Magnesium deficiency in Western societies has been linked to insulin resistance [22], [23], type II diabetes [24], [25], metabolic syndrome [26], coronary heart disease [27], colorectal cancer [28], and colorectal adenoma [29]. Similar to the relationships between obesity and most chronic diseases, systemic inflammation may be a unifying pathway by which magnesium deficiency contributes to such a broad range of morbidity [19], [30]. Chronic inflammation may also play a key role in the progression from normal tissue to prostatic intraepithelial neoplasia (PIN) and prostate cancer [31], and it is also possible that the elevated inflammatory response associated with magnesium deficiency is dependent on concurrent calcium levels [19].

The purpose of this study is to investigate the association between serum magnesium with prostatic intraepithelial neoplasia (PIN), low-grade prostate cancer, and high-grade prostate cancer. We further investigated the interaction between magnesium and calcium levels on aggressive or high-grade prostate cancer risk, hypothesizing that inadequate serum magnesium levels reflected by a high serum ratio of calcium to magnesium will be associated with more aggressive prostate cancer.

## Materials and Methods

### Study Population

The Nashville Men's Health Study (NMHS) utilizes a multi-centered, rapid-recruitment protocol to collect clinical, biological, behavioral, and body measurement data from men scheduled for diagnostic prostate biopsy. All participants provided written informed consent in accordance with the Vanderbilt University IRB. Men scheduled for a diagnostic prostate biopsy between 2002 and 2008 at a Vanderbilt University Medical Center (Nashville, TN), the Tennessee Valley Veteran's Administration Hospital (Nashville, TN), or a large private urology practice in Nashville, were approached for recruitment. Eligible participants were 40 years of age or older and had no prior prostate cancer diagnosis. Approximately 95% of eligible men approached for recruitment agree to participate, and the study population included 2,100 eligible consenting subjects. Pathology data were analyzed from two labs, but over 90% of patients were diagnosed at a large community urologic partnership with one on-site pathologist. We have compared in a sub-sample of 60 men pathology scoring at biopsy from pathology after gland removal (the definitive staging) and found similar biopsy Gleason score categories.

### Biomarker Sub-Study

In 2009, a sub-study of 503 NMHS participants was developed for translational investigations of promising biomarkers hypothesized to be associated with prostate cancer risk. We included all 137 available PIN cases available to us at that time through recruitment, and PIN cases served as the age index for frequency matching. We then randomly selected from our available recruitment 101 high-grade prostate cancer cases (Gleason = 7(4+3 only), 8, 9, or 10) and 100 low-grade prostate cancer cases (Gleason = 6) such that the PIN, low-grade, and high-grade cases had an age distribution between 50–80 years and had a similar age distribution using 5-year age categories. We also randomly selected 165 biopsy-negative controls using identical age criteria from approximately 900 candidates, although we oversampled controls less than age 55 through random selection to support the analysis of younger men with fewer cancer outcomes.

### Data Collection

Measures of body size and weight were collected by a trained research staff member at the time of recruitment. Participants wore a hospital gown or other light clothing, and did not wear shoes. Chart review included age, race, PSA history, and prostate needle-biopsy result (cancer, PIN, negative, or a suspicious, atypical, or other lesion). Gleason scores at biopsy were also ascertained for subjects diagnosed with cancer following pathology review of the biopsy specimen. Prostate volume (cm^3^) was measured by transrectal ultrasound (TRUS) during the prostate biopsy procedure. Family history of prostate cancer was ascertained from the surgical chart and by a structured research questionnaire administered to each participant upon recruitment.

As a part of the pre-biopsy clinic visit, all NMHS participants are instructed to make a list of all current medications at home or to bring their medications to this clinic visit. Prior to biopsy, the surgeon reviews all survey responses with the participant to confirm that the subject has been taking the listed drugs. Additionally, the surgeon makes specific queries to the subject for drugs such as aspirin or Warfarin that might affect bleeding or clotting during the biopsy procedure. All prescription and non-prescription drugs were abstracted from the medical record following consent from the participant. Drug categories were developed to represent men receiving treatment for cardiovascular disease, diabetes, BPH, or taking a non-steroidal anti-inflammatory agent. Drugs formulated as a combination of two or more active drugs were classified by each of the component drugs.

### Biomarker Assays

Serum concentration of magnesium and calcium were determined by standard analytic method on the Beckman DXC 800 chemistry analyzer provided by the Vanderbilt Pathology Laboratory Services with an intra-assay coefficient of variation of 2.0. Several biomarkers associated with obesity were also analyzed. All study biomarker assays include a standard set of blank and positive controls and were performed in a single batch and blind to case-control status.

### Data Analysis

Among 503 participants, we dropped these participants (1 high-grade cancer, 1 PIN, 1 control) for missing magnesium levels; (1 control, 1 low-grade cancer, 1 PIN) for missing calcium levels; and (1 PIN, 1 high-grade cancer) for missing both Mg and Ca. We also dropped 1 PIN case with severe hypocalcemia (2.5 ng/ml). Thus, the final analytic study population included 494 participants (99 high-grade cancer and 99 low-grade cancer, 133 PIN, and 163 negatives). Analyses that controlled for WHR were limited to 493 participants with WHR.

The current molecular sub-study was independently funded to test our *a priori* hypothesis, as stated from our grant, that low serum magnesium levels are associated with increased risk of PIN and prostate cancer, perhaps more so among men with elevated calcium levels. Thus, our primary analyses investigated the associations between serum magnesium and calcium/magnesium ratio and risk of PIN and prostate cancer, whereas all other analyses were conducted to support the primary analysis or to explore nature of our primary findings. Because we also proposed the direction of the association in the *a priori* hypothesis, one-sided tests were performed for primary hypotheses.

Prior to testing our primary hypothesis, preliminary analyses compared mean magnesium and calcium levels across age categories, demographics, obesity measures, PSA levels, prostate volume, NSAID use, treatment for BPH, CVD, hypercholesterolemia, or diabetes. The distributions of magnesium, calcium, and the ratio of calcium to magnesium ratio (Ca/Mg) were approximately normally distributed and therefore were not transformed for analysis. Our primary analyses compared mean magnesium and calcium levels between cancer, PIN, and control groups. We report mean values adjusted for WHR, diabetes treatment, CVD treatment, and race in a linear model after finding that these factors were significantly associated with either calcium or magnesium levels, orCa/Mg (p<0.05). We also adjusted for age to accommodate potential residual confounding not addressed through the frequency matching of cases and controls for the biomarker sub-study. Logistic regression was used to calculate odds ratios (ORs) and 95% confidence intervals summarizing the association between magnesium, calcium, or Ca/Mg with each case outcome in separate models adjusting for age or for age, WHR, race, and treatment for diabetes and CVD. Magnesium, calcium, and Ca/Mg were scaled as continuous variables or categorized at tertiles of the control-group distribution. Tests for interaction were determined by cross-product term between magnesium or calcium and the covariates of interest in the presence of each main-effect and other covariates.

## Results

Controls, PIN cases, and cancer cases had similar distributions for age, race, and family history of prostate cancer (**Table 1**). Serum levels of magnesium, calcium, and Ca/Mg averaged 2.14 ng/ml, 9.72 ng/ml, and 4.58, respectively. Magnesium levels were not significantly correlated with calcium levels overall (r = −0.06, p = 0.17), or within controls (r = 0.06, 0.48), or low-grade (r = −0.05, p = 0.62) or high-grade (r = −0.07, 0.52) cancer cases.There was a significant but weak inverse correlation between magnesium and calcium levels among PIN cases (r = −0.20, p = 0.02).

**Table 1:** Study population description.

		Controls		PIN		Low-Grade			High-Grade	
Factor		mean	sd	mean	sd	mean	sd	mean		sd
Age (yrs)		66.7	7.7	65.6	6.5	67.0	7.4	67.9		6.8
	Level	n	%	n	%	n	%	n		%
	50–54	10	6.2%	5	3.8%	7	7.0%	3		3.1%
	55–59	20	12.4%	26	19.6%	9	9.0%	9		9.2%
	60–64	31	19.1%	24	18.1%	15	15.2%	16		16.1%
	65–69	34	20.9%	31	23.3%	28	28.3%	31		31.6%
	70–74	36	22.1%	39	29.3%	20	20.2%	20		20.2%
	75–79	32	19.6%	8	6.0%	20	20.2%	20		20.2%
Family history	Yes	29	17.8%	28	21.0%	16	16.2%	13		13.1%
	Unsure	84	51.5%	55	41.4%	59	59.6%	61		61.6%
	No	50	30.7%	50	37.6%	24	24.2%	25		25.3%
Race/Ethnicity	White	147	90.2%	120	90.2%	88	88.9%	88		88.9%
	Black	15	9.2%	13	9.8%	10	10.1%	11		11.1%
	Asian or Hispanic	1	0.6%	0	0%	1	1.0%	0		0%

Magnesium levels were significantly lower among non-white participants, as well as participants being treated for diabetes or CVD (**Table 2**). However, only 16 non-white subjects were being treated for diabetes. Calcium and the Ca/Mg were also significantly associated with treatment for diabetes, and the Ca/Mg ratio was significantly lower among whites compared to non-whites. Furthermore, serum calcium and the Ca/Mg ratio were significantly higher with a higher WHR. In contrast, magnesium, calcium, and Ca/Mg were not significantly associated with current use of steroid reductase inhibitors and/or alpha blockers, PSA levels, prostate size, BMI, or other factors considered. Thus, final analytic models did not adjust for current use of steroid reductase inhibitors and/or alpha blockers, PSA and prostate size, BMI, or volume.

**Table 2:** Association between Mg, Ca, and Ca/Mg with study population characteristics.

Factor	Level	n*	Mg	Ca	Ca/Mg
Age (yrs)	50–54	25	2.25	9.63	4.30
	55–59	64	2.12	9.88	4.73
	60–64	86	2.17	9.80	4.56
	65–69	124	2.16	9.71	4.59
	70–74	115	2.12	9.67	4.61
	75–79	80	2.14	9.65	4.54
		P** =	0.19	0.43	0.16
Race/Ethnicity	Non-white	51	2.03	9.82	4.88
	White	443	2.16	9.71	4.55
		P =	<0.01	0.36	<0.01
Family History	Yes	86	2.17	9.71	4.52
	Unsure	259	2.13	9.72	4.64
	No	149	2.18	9.74	4.53
		P =	0.06	0.95	0.16
BMI (kg/m^2^)	16–19	3	2.05	9.73	4.77
	20–24	76	2.12	9.63	4.61
	25–29	248	2.15	9.69	4.56
	30–34	125	2.16	9.81	4.59
	35–52	42	2.14	9.82	4.64
		P =	0.69	0.51	0.93
Height (cm)	157–170	129	2.14	9.65	4.55
	171–174	109	2.15	9.79	4.63
	175–178	131	2.16	9.64	4.51
	179–192	125	2.14	9.83	4.65
		P =	0.94	0.14	0.32
Waist (cm)	73–97	127	2.16	9.61	4.51
	98–104	120	2.14	9.64	4.57
	105–111	123	2.15	9.82	4.63
	112–145	123	2.15	9.83	4.63
		P =	0.92	0.06	0.45
WHR	0.780–0.978	125	2.15	9.60	4.52
	0.979–1.022	125	2.18	9.67	4.48
	1.023–1.061	119	2.14	9.89	4.68
	1.062–1.230	124	2.12	9.76	4.68
		P =	0.22	0.04	0.03
PSA (ng/ml)	0.2–3.9	85	2.15	9.72	4.55
	4.0–9.9	321	2.16	9.73	4.57
	10.0–334	84	2.12	9.73	4.67
		P =	0.43	0.97	0.38
Volume (mls)	10–9	190	2.13	9.71	4.62
	40–59	161	2.17	9.75	4.54
	60–27	127	2.15	9.74	4.60
		P =	0.29	0.91	0.49
NSAIDs (regular use)	Yes	225	2.16	9.79	4.60
	No	269	2.14	9.67	4.57
		P =	0.51	0.08	0.70
Treatment for					
BPH	Yes	143	2.15	9.69	4.57
	No	351	2.15	9.74	4.59
		P =	0.99	0.57	0.79
CVD	Yes	300	2.13	9.70	4.63
	No	194	2.18	9.76	4.52
		P =	0.02	0.42	0.07
Hyperlipidemia	Yes	204	2.15	9.77	4.61
	No	290	2.15	9.70	4.57
		P =	0.88	0.25	0.57
Diabetes	Yes	78	2.04	9.90	4.94
	No	416	2.17	9.69	4.52
		P =	<0.01	0.03	<0.01

*missing values: waist (n = 1), WHR (n = 1), PSA (n = 4), volume (n = 16).

**p-value to test for one-way ANOVA testing for differences in Mg, Ca, or Ca/Mg between levels of each factor.

Magnesium levels were approximately 5% lower among high-grade cancer cases compared to controls (p = 0.04) or PIN cases (p = 0.03) after adjusting for age, WHR, race, and treatment for diabetes and CVD (**Table 3**). Accordingly, the Ca/Mg ratio was significantly higher among high-grade cases compared to controls (p = 0.01) and PIN cases (p = 0.05). The Ca/Mg ratio was also higher among high-grade cancer cases compared to low-grade cases (p = 0.05). In contrast, serum calcium levels were somewhat higher among high-grade prostate cancer cases, but differences in calcium levels between groups were not statistically significant.

**Table 3:** Adjusted Mean Mg (ng/ml), Ca (ng/ml), or Ca/Mg Levels, PIN, and Prostate Cancer.

		Mg (ng/ml)		Ca (ng/ml)		Ca/Mg	
	n	Mean	95% CI	Mean	95% CI	Mean	95% CI
Age-Adjusted							
Negative-Control	163	2.16	2.13, 2.20	9.70	9.57, 9.82	4.52	4.42, 4.63
PIN	133	2.18	2.14, 2.22	9.73	9.59, 9.87	4.52	4.41, 4.64
Low-Grade cancer	99	2.14	2.10, 2.19	9.66	9.50, 9.82	4.57	4.44, 4.71
High-Grade cancer	98	2.09∧	2.04, 2.13	9.82	9.66, 9.98	4.78∧∧	4.65, 4.92
Fully-Adjusted*							
Negative-Control	163	2.09	2.04, 2.13	9.81	9.64, 9.98	4.75	4.62, 4.89
PIN	133	2.09	2.04, 2.14	9.87	9.68, 10.06	4.79	4.64, 4.94
Low-Grade cancer	99	2.07	2.02, 2.13	9.76	9.56, 9.95	4.78	4.62, 4.94
High-Grade cancer	98	2.03**	1.97, 2.08	9.91	9.72, 10.10	4.96***	4.81, 5.11

∧ High-grade cancer vs. (negative-controls: p = 0.01), (PIN: p<0.01), or (low-grade cancer: p = 0.09).

∧∧ High-grade cancer vs. (negative-controls: p<0.01), (PIN: p<0.01), or (low-grade cancer: p = 0.03).

*Adjusted for age (continuous), treatment for diabetes (Yes/No), treatment for CVD (Yes/No), WHR (categorized at quartiles), and race (white, non-white).

**High-grade cancer vs. (negative-controls: p = 0.04), (PIN: p = 0.03), or (low-grade cancer: p = 0.13).

***High-grade cancer vs. (negative-controls: p = 0.01), (PIN: p = 0.05), or (low-grade cancer: p = 0.05).

No other differences were significant at p<0.05.Additional adjustment for family history did not change these results.

Similarly, increasing magnesium levels were associated with a lower likelihood of being diagnosed with high-grade prostate cancer. For example, magnesium levels in the highest tertile were associated with an approximate 48% reduction in risk (OR = 0.52, (0.26, 1.02; p-trend = 0.04). This association became stronger after additional control for calcium (OR = 0.48 (0.24–0.96; p-trend = 0.03) (**Table 4**). While high-grade cancer was not associated with calcium levels, the Ca/Mg ratio was associated with an increasing trend for greater risk of high-grade prostate cancer. Furthermore, Ca/Mg as a continuous variable was significantly associated with high-grade disease (OR = 2.81 (1.24, 6.36), adjusted for magnesium, calcium, and other covariates). **Table 5** summarizes the relationship between magnesium levels and high-grade disease across calcium levels, and suggests that higher magnesium levels may lower the risk of high-grade prostate cancer among men with high calcium levels (OR = 0.48 (0.23, 1.00)).

**Table 4:** Association between Mg, Ca, or Ca/Mg with PIN and Prostate Cancer.

		Scale	N (Case/Ctl)	OR	95% CI	OR∧	95% CI
PIN	Mg (ng/ml)	continuous	133/163	1.07	0.36, 3.21	1.03	0.33, 3.17
		<2.1	30/43	1.0	ref	1.0	ref
		2.1–2.3	58/62	1.26	0.69, 2.32	1.24	0.67, 2.29
		>2.3	45/58	0.97	0.52, 1.82	0.95	0.50, 1.81
	Ca (ng/ml)	continuous	133/163	1.08	0.81, 1.45	1.08	0.80, 1.45
		<9.2	37/54	1.0	ref	1.0	ref
		9.2–9.9	47/49	1.31	0.72, 2.38	1.29	0.70, 2.35
		>9.9	49/60	1.24	0.69,2.23	1.23	0.68, 2.21
	Ca/Mg	continuous	133/163	1.10	0.75, 1.61	1.13	0.53, 2.41
		<4.17	40/52	1.0	ref	1.0	ref
		4.17–4.67	47/55	1.16	0.65, 2.07	1.08	0.51, 2.30
		>4.67	46/56	1.28	0.71, 2.32	1.46	0.48, 4.47
Low-Grade	Mg (ng/ml)	continuous	99/163	0.71	0.22, 2.31	0.58	0.17, 1.98
		<2.1	30/43	1.0	ref	1.0	ref
		2.1–2.3	33/62	0.79	0.41, 1.51	0.72	0.37, 1.41
		>2.3	36/58	0.92	0.47, 1.81	0.81	0.40, 1.61
	Ca (ng/ml)	continuous	99/163	0.87	0.62, 1.22	0.89	0.63, 1.25
		<9.2	23/54	1.0	ref	1.0	ref
		9.2–9.9	47/49	2.32	1.20, 4.50	2.44	1.25, 4.77
		>9.9	29/60	1.02	0.51, 2.03	1.07	0.53, 2.14
	Ca/Mg	continuous	99/163	1.05	0.69, 1.61	1.22	0.55, 2.71
		<4.17	33/52	1.0	ref	1.0	ref
		4.17–4.67	27/55	0.75	0.39, 1.45	0.71	0.30, 1.68
		>4.67	39/56	1.00	0.53, 1.90	1.17	0.36, 3.77
High-Grade	Mg (ng/ml)	continuous	98/163	0.31	0.10, 0.97	0.26	0.09, 0.85
		<2.1	38/43	1.0	ref	1.0	ref
		2.1–2.3	37/62	0.73	0.40, 1.36	0.70	0.38, 1.30
		>2.3	23/58	0.52	0.26, 1.02	0.48	0.24, 0.96
	Ca (ng/ml)	continuous	98/163	1.16	0.85, 1.59	1.19	0.86, 1.63
		<9.2	29/54	1.0	ref	1.0	ref
		9.2–9.9	36/49	1.43	0.75, 2.75	1.58	0.81, 3.06
		>9.9	33/60	1.01	0.53, 1.95	1.04	0.54. 2.02
	Ca/Mg	continuous	98/163	1.68	1.11, 2.53	2.81	1.24, 6.36
		<4.17	22/52	1.0	ref	1.0	ref
		4.17–4.67	29/55	1.22	0.61, 2.41	1.12	0.48, 2.66
		>4.67	47/56	1.84	0.94, 3.61	2.04	0.64, 6.58

Adjusted for age, treatment for diabetes, treatment for CVD, WHR, and race.

∧ also adjusted for magnesium, calcium, or magnesium and calcium, as appropriate.

**Table 5:** Joint effects of Calcium and Magnesium with High-grade Prostate Cancer.

		n	Low Mg (<2.2 ng/ml)		n	High Mg (> = 2.2 ng/ml)	
		Case/Control	OR	95% CI	Case/Control	OR	95% CI
Calcium	High (> = 9.4 ng/ml)	37/40	1.0	Ref	18/45	0.48	0.23, 1.00
	Low(<9.4 ng/ml)	25/37	0.69	0.34, 1.38	18/41	0.52	0.24, 1.12

p-values: Ca: p = 0.58, Mg: p = 0.07, p-interaction = 0.40.

Adjusted for age, treatment for diabetes, treatment for CVD, WHR, and race.

## Discussion

We found serum magnesium levels, and the ratio of calcium-to-magnesium (Ca/Mg), was significantly associated with high-grade prostate cancer. Calcium levels alone, in contrast, were not consistently associated with prostate cancer or PIN. Our analyses also suggested that increased blood magnesium levels have an effect that may be at least partially dependent on calcium levels toward the pathogenesis for high-grade prostate cancer.

Calcium has long held an interest in cancer with essential messenger roles regulating cell cycle proliferation and apoptosis [32]–[34]. Calcium consumption might also reduce the production of 1,25-dihydroxyvitamin D (1,25(OH)2D; calcitriol), the hormonal form of vitamin D [35]. However, studies of dietary calcium intake in relation to aggressive prostate cancer have produced mixed results [2], with little evidence of an association in the Melbourne Collaborative Cohort Study [6], the Calcium Polyp Prevention Study [3], the Prostate, Lung, Colorectal and Ovarian Cancer Screening Trial [4], or a recent prospective analysis of blood calcium levels [5]. In contrast, several prospective studies have suggested that greater intake of calcium [2] or a higher level of serum calcium [14], [15] is associated with aggressive, poorly differentiated, lesions or fatal prostate cancer. Our analysis may be the first to suggest that the relationship between calcium and prostate cancer depends, at least to some degree, on the counter-effects of magnesium, and provides one possible explanation for some inconsistency in the previous studies.

Magnesium plays an essential role in DNA repair, cell differentiation and proliferation, apoptosis, and angiogenesis [17], [19], [36]. Magnesium deficiency is also linked to the inflammatory response [19] and oxidative stress [37], while magnesium supplementation, in contrast, improves insulin sensitivity and reduces insulin levels [23] [22]. Given the broad range of biological functions dependent on magnesium, it is highly plausible that magnesium deficiency may affect multiple pathways toward tumorigenesis across the body. For example, a recent study found mice transplanted with Lewis Lung Carcinoma and receiving a low-Mg diet had a significant 70% reduction in primary tumor growth, but also had a higher metastatic potential [38]. Interestingly, we found low serum Mg was only associated with an increased risk of high-grade prostate cancer, but not PIN or low-grade prostate cancer, perhaps consistent with the higher metastatic potential found in the prior animal studies. Magnesium acts as a physiologic antagonist to ionized calcium [17], and a low ionized magnesium level may further potentate the activity of ionized calcium [17]. We found the Ca/Mg ratio was associated with high grade prostate cancer after adjusting for magnesium and calcium levels, and there was a somewhat stronger, although not significantly stronger, association between magnesium and high-grade prostate cancer among men with higher serum calcium.

Thus, our data suggest that magnesium levels may influence the progression of prostate lesions to a higher grade. Migration studies further reveal a substantial increase in the incidence of advanced prostate cancer as men move from Eastern Asia to the West [29], [39], while the age-specific prevalence of indolent and early-stage prostate cancer lesions detected at autopsy are relatively uniform across countries [40]. Magnesium intake is similar between the East and West, however the ratio of dietary calcium to magnesium intake is much higher (2.8) in the US population than (1.6) in East Asia [29], perhaps contributing to international differences in prostate cancer risk described above, as well as colorectal cancer risk [29]. We found previously that the calcium/magnesium intake ratio modified the association between calcium intake and magnesium intake on the risk of colorectal adenoma [29]. Similarly, we found in our current prostate cancer study that African Americans had a significantly higher Ca/Mg serum ratio than whites, and our findings provide a possible underlying mechanism for the racial disparity in fatal prostate cancer.

There are several strengths to this analysis, including the opportunity to evaluate the potential for detection biases related to differences in PSA,prostate volume. Blood was collected prior to diagnosis and treatment, and body size measures were ascertained by trained staff. One concern is that the temporal relationship between magnesium, or the calcium/magnesium ratio, and carcinogenesis cannot be conclusively determined. Although we cannot exclude the possibility that the alterations in levels of magnesium and calcium are an effect rather than a cause of prostate cancer, previous animal studies suggest that our associations are not an effect of prostate cancer. Furthermore, prior studies in humans found lower levels of magnesium (thus, higher serum calcium/magnesium ratio) lead to inflammation and insulin resistance which have been linked to progression of prostate cancer. In our study, we only found significant differences between negative controls with high-grade cancer, but not PIN or low-grade cancer. This argues that lower levels of magnesium (or high calcium/magnesium ratio) are not simply a consequence of cancer, but does not remove the possibility that high-grade cancer in some way affects blood Mg levels. There is a somewhat higher probability that high-grade prostate cancer has metastasized to the bone, and bone provides a repository for circulating calcium and magnesium. However, clinical chart review from 189 prostate cancer cases from this study found that no evidence of metastatic disease or lymph node involvement at diagnosis. Nevertheless, prospective studies are warranted to confirm our novel findings. Although men with prior BPH surgery were not eligible for this analysis and we found no association between current BPH treatment with Mg or Ca, we cannot eliminate the possibility that a presumably small number of past finasteride user affected our results. The difference in Ca/Mg serum ratio between whites and African Americans needs to be interpreted with caution because this was a secondary analysisbased on only 50 African American participants.

Our finding indicates that higher blood magnesium levels are associated with a lower risk of high-grade prostate cancer. The lower ratio of calcium to magnesium was also associated with high-grade prostate cancer, suggesting the interaction between magnesium and calcium plays a role in the pathogenesis and progression of this disease to a more clinically relevant phase. These findings, if confirmed, may provide a new avenue for the personalized prevention or adjuvant care of prostate cancer.
